# Chlorhexidine gluconate, its properties and applications in endodontics

**Published:** 2008-01-10

**Authors:** Zahed Mohammadi

**Affiliations:** 1 *Department of Endodontics, Dental School, Shahid Sadoughi University of Medical Sciences, Yazd, Iran.*

**Keywords:** Antibacterial agents, Antifungal agents, Chlorhexidine, Irrigation

## Abstract

The major objective in endodontic therapy is to disinfect the entire root canal system. This requires that the pulpal content be eliminated as sources of infection. This goal may be accomplished by mechanical instrumentation and chemical irrigation, in conjunction with medication of the root canal between treatment sessions. Microorganisms and their by-products are considered to be the major cause of pulpal and periradicular pathosis. In order to reduce or eliminate bacteria from the root canal system, various irrigants have been used during treatment. Chlorhexidine is a cationic solution which can be used during treatment. It has a wide range of antimicrobial activity. Furthermore, because of its cationic structure, chlorhexidine has a unique property named substantivity. The purpose of this paper is to review different aspects of chlorhexidine in endodontics.

## Introduction

The essential role of microorganisms in development and perpetuation of pulpal and periapical diseases have clearly been demonstrated in animal models and human studies (1-3). Elimination of microorganisms from infected root canals is a complicated task. Numerous measures have been described to reduce number of root canal microorganisms, including the use of various instrumentation techniques, irrigation regimens and intra-canal medicaments. There is no solid evidence in the literature that mechanical instrumentation alone results in a bacteria-free root canal system. Considering the complex anatomy of root canal pulp space (4), this is not surprising. On the contrary, there is in vitro and clinical evidence that mechanical instrumentation leaves significant portion of the root canal walls untouched (5) and complete elimination of bacteria from the root canal by cleaning the root canal by instrumentation alone is unlikely (6). It is assumed, but not demonstrated, that any pulp tissue left in the root canals can serve as bacterial nutrient. Furthermore, tissue remnants also inactivate or reduce the antimicrobial effects of root canal irrigants and medicaments. Therefore some sort of irrigation/ disinfection is necessary to remove tissue from the root canals and to kill microorganisms simultaneously. Simply, chemical treatment of the root canal can be arbitrarily divided into irrigants, rinses, and inter-visit medicaments. Chlorhexidine is used widely as an endodontic irrigant and medicament. However, there is no adequate evidence on different aspects of Chlorhexidine (CHX). The purpose of this paper is to review different aspects of CHX.

## Structure and mechanism of action

CHX is a synthetic cationic bis-guanide consists of two symmetric 4-cholorophenyl rings and two biguanide groups connected by a central hexamethylene chain (7). CHX is a positively charged hydrophobic and lipophilic molecule that interacts with phospholipids and lipopolysaccharides on the cell membrane of bacteria and then enters the cell through some type of active or passive transport mechanism (8). Its efficacy is due to the interaction of positive charge of the molecule and negatively charged phosphate groups on the microbial cell walls (9), thereby altering the cells' osmotic equilibrium. This increases the permeability of the cell wall, which allows the CHX molecule penetrate into the bacteria. CHX is a base and is stable as a salt. The most common oral preparation, chlorhexidine gluconate, is water-soluble and at physiologic pH, readily dissociates and releases the positively charged CHX component (7). At low concentration (0.2%), low molecular weight substances specifically potassium and phosphorous will leak out. On the other hand, at higher concentration (2%), CHX is bactericidal; precipitation of cytoplasmic contents occurs resulting in cell death (9).

## Antibacterial activity

Delany *et al.* (10) evaluated the 0.2% CHX gluconate on infected root canals. Bacteriologic samples were obtained before, during, immediately after and 24 hours after instrumentation, irrigation, and medication either with CHX gluconate or with sterile saline. There was a highly significant reduction in microorganisms in the CHX-treated specimens after the instrumentation and irrigation procedures. Basson and Tait (11) compared the effectiveness of calcium hydroxide, iodine potassium iodide (IKI) and a CHX solution in disinfecting *Actinomyces (A) israelii*-infected root canal walls and dentinal tubules *in vitro*. The root canals were exposed to either IKI, calcium hydroxide or 2% CHX for periods of 3, 7 and 60 days. CHX was the only disinfectant that was able to eliminate *A. israelii* from all the samples at all periods while 25% of the specimens treated with IKI and 50% of the specimens treated with calcium hydroxide still had viable *A. israelii* after treatment. Oncag *et al.* (12) evaluated the antibacterial properties of 5.25% sodium hypochlorite (NaOCl), 2% CHX and 0.2% CHX plus 0.2% cetrimide (Cetrexidin) after 5 min and after 48 h in extracted human teeth, whose canals were infected by *Enterococcus faecalis*. The 2% CHX and Cetrexidin were significantly more effective on *E. faecalis* than the 5.25% NaOCl at both time periods. Gomes *et al.* (13) and Vianna *et al*. (14) investigated *in vitro* the antimicrobial activity of three concentrations (0.2%, 1% and 2%) of two forms of CHX (gel and liquid) against endodontic pathogens and compared the results with the ones achieved by five concentrations of NaOCl (0.5%, 1%, 2.5%, 4% and 5.25%).

Both 2% gel and liquid formulation of CHX eliminated *Staphylococcus aureus* and *Candida albicans* in 15 seconds, whereas the gel formulation killed *E. faecalis* in 1 min. All tested irrigants eliminated *Porphyromonas endodontalis*, *Porphyromonas gingivalis*, and *Prevotella intermedia* in 15 seconds. The time required to eliminate all microorganisms was the same for 5.25% NaOCl. The timing required for 1.0% and 2.0% CHX liquid to eliminate all micro organisms was the same required for 5.25% NaOCl (14). The antimicrobial activity is related to type, concentration, and presentation form of the irrigants as well as the microbial susceptibility.

Zamany *et al*. (15) examined addition of a 2% CHX rinse to the conventional treatment protocol on the successful disinfection of the root canal system. Results showed that cultivable bacteria were retrieved at the conclusion of the first visit in 1 out of CHX cases, whereas in the control group 7 out of 12 cases showed growth. This difference was significant. Siqueira *et al.* (16) compared the effectiveness of 2.5% NaOCl and 0.12% CHX as irrigants in reducing the cultivable bacteria in infected root canals of teeth with apical periodontitis. They found that both solutions revealed comparable results as to the bacterial elimination from infected root canals and suggested that both can be used as irrigants. In a randomized clinical trial, Manzur *et al.* (17) assessed the antibacterial efficacy of intracanal medication with calcium hydroxide, 2% CHX gel, and a combination of both [Ca(OH)_2_/CHX] in teeth with chronic apical periodontitis. Bacteriological samples obtained from the operative field and the root canals before and after instrumentation in the first treatment session, and after medication in the second session one week later. They concluded that the antibacterial efficacy of Ca(OH)_2_, CHX, and Ca(OH)_2_/CHX was comparable. Zerella *et al.* (18) investigated the effect of a slurry of Ca(OH)_2_ mixed in aqueous 2% CHX versus aqueous Ca(OH)_2_ slurry alone on the disinfection of the pulp space of failed root-filled teeth during endodontic retreatment. Of the total sample population, 12 of 40 (30%) were positive for bacteria before root filling. The control medication disinfected 12 of 20 (60%) teeth including 2 of 4 teeth originally diagnosed with *Enterococci*. The experimental medication resulted in disinfected 16 of 20 (80%) teeth at the beginning of the third appointment. None of the teeth originally containing *Enterococci* showed remaining growth. They concluded that canal dressing with a mixture of 2% CHX and Ca(OH)_2_ slurry is as efficacious as aqueous Ca(OH)_2_ on the disinfection of failed root-filled teeth. Ercan *et al.* (19) evaluated the antibacterial activity of 2% CHX and 5.25% sodium hypochlorite in infected root canals of incisors and premolars. They concluded that both CHX and sodium hypochlorite were significantly effective to reduce the microorganisms in the teeth with necrotic pulp, periapical pathologies, or both, and could be used successfully as an irrigant solution. Tanomaru *et al.* (20) evaluated the effect of biomechanical preparation with 5% NaOCl, 2% CHX and physiological saline irrigating solutions and calcium hydroxide dressing in dog root canals containing bacterial endotoxin. They found that biomechanical preparation with the irrigating solutions did not inactivate the effects of the endotoxin but the calcium hydroxide intracanal dressing did appear to inactivate the effects induced by the endotoxin *in vivo*. Another interesting topic is the additive effect of CHX and hydrogen peroxide. Heling and Chandler (21) studied the antimicrobial effect of irrigant combinations within dentinal tubules in vitro against *E. faecalis* and found that a specific combination of 3% hydrogen peroxide (H_2_O_2_) and CHX was superior in its antibacterial activity in dentine compared with other regimens such as CHX alone and NaOCl. Steinberg *et al.* (22) challenged *E. faecalis* suspensions in trypticase soy broth (a culture medium rich in peptides) with various combinations of CHX and H_2_O_2_. The experiments demonstrated that the combination of the two substances totally killed *E. faecalis* in concentrations much lower than each component alone. According to that study, the bactericidal effect of CHX is due to its ability of denaturating the bacterial cell wall while forming pores in the membrane, while H_2_O_2_ is effective against intracellular organelles such as DNA. Although the exact synergistic mechanism of CHX and H_2_O_2_ is not known, it can be postulated that the exposure of bacteria to CHX leads to a more permeable cell wall that H_2_O_2_ can penetrate easily and hence damage the intracellular organelles (22).

On the whole, although studies comparing the antibacterial effect of CHX and NaOCl have produces somewhat conflicting results, it seems that when used in identical concentrations, their antibacterial effect *in vitro* (infected dentine) and *in vivo* (in the root canal system) is similar.

## Antifungal activity

Fungi constitute a small part of the oral microbiota. The largest proportion of the fungal microbiota is made up of *Candida* species. *Candida albicans* is the fungal species most commonly detected in the oral cavity of both healthy (30-45%) and medically compromised (95%) individuals (23). Fungi have occasionally been found in primary root canal infections, but they seem to be more common in the root canals of obturated teeth with failed treatment (23). Overall, the occurrence of yeasts reported in infected root canals varies between 1% and 17% (24).

Because fungi may be involved in cases of persistent and secondary infections associated with recalcitrant periradicular lesions, the spectrum of antimicrobial activity of endodontic medicaments and irrigants should include these microorganisms. Thus, strategies with medicaments that have antifungal effectiveness may assist in the successful management of persistent or secondary endodontic infections caused by fungi (23, 24).

To improve antisepsis in a one-appointment regime, it has been suggested to rinse/soak the canals with CHX or IPI solutions following irrigation with sodium hypochlorite. Aqueous CHX solution has a wide-spectrum antimicrobial activity at low concentrations, and is especially effective against *C. albicans*. Furthermore, it binds to surrounding tissues to be released again slowly over extended periods of time, a phenomenon called substantivity. Interestingly, it appears that chlorhexidine can efficiently inhibit the initial adherence and perhaps further accumulation and biofilm formation of yeasts and other microorganisms. A recent clinical study has shown that canals that received a final rinse with a 2% CHX solution were significantly more often free of cultivable microorganisms than controls irrigated with sodium hypochlorite alone (23,24).

Sen *et al.* (25) evaluated the antifungal properties of 0.12% CHX, 1% NaOCl, and 5% NaOCl against *Candida albicans* using cylindrical dentine tubes. They found that *C. albicans* to be more resistant in the presence of smear layer than in the absence of smear layer. When smear layer was absent, NaOCl started to display antifungal activity after 30 minutes. Waltimo *et al.* (26) evaluated the susceptibility of 7 strains of *C albicans* to 4 disinfectants: IKI, CHX acetate, sodium hypochlorite, and calcium hydroxide. In addition, all possible pairs of the disinfectants were tested to compare the effect of the combination and its components. *C. albicans* cells were highly resistant to calcium hydroxide. Sodium hypochlorite (5% and 0.5%) and IKI killed all yeast cells within 30 s, whilst CHX acetate (0.5%) showed complete killing after 5 min. Combinations of disinfectants were equally or less effective than the more effective component. All *C. albicans* strains tested showed similar susceptibility to the medicaments tested. Siqueira *et al.* (27) evaluated the effectiveness of four intracanal medications in disinfecting the root dentine in bovine teeth experimentally infected with *C. albicans*. Infected dentine cylinders were exposed to four different medications: calcium hydroxide/glycerin; calcium hydroxide/0.12% CHX; calcium hydroxide/camphorated para-monochlorophenol/glycerin; and 0.12% CHX/ zinc oxide. Results showed that the specimens treated with calcium hydroxide/camphorated paramonochlorophenol/glycerin paste or with CHX/zinc oxide paste were completely disinfected after 1 h of exposure and calcium hydroxide/glycerin paste consistently eliminated *C. albicans* infection after 7 d of exposure. Calcium hydroxide mixed with CHX was ineffective in disinfecting dentine even after 1 w. In another study, Siqueira *et al.* (28) investigated the antifungal ability of several medicaments against *C. albicans*, *C. glabrata*, *C. guilliermondii*, *C. parapsilosis*, and *S. cerevisiae*. Whereas the paste of calcium hydroxide in CPMC/glycerin showed the most pronounced antifungal effects, calcium hydroxide in glycerin or CHX and CHX in detergent also showed antifungal activity that was much lower than the paste of calcium hydroxide in CPMC/glycerin. Ferguson *et al.* (29) sought to determine the *in vitro* susceptibility of *C. albicans* to various irrigants and medicaments. The minimum inhibitory concentrations of NaOCl, hydrogen peroxide, CHX digluconate, and aqueous calcium hydroxide were determined. Their results revealed that NaOCl, hydrogen peroxide, and CHX digluconate were effective against *C. albicans* even when significantly diluted. Aqueous calcium hydroxide had no activity.

## CHX and biofilms

The term biofilm was introduced to designate the thin-layered condensations of microbes that may occur on various surface structures in nature. Free-floating bacteria existing in an aqueous environment, so-called planktonic microorganisms are a prerequisite for biofilm formation (30). Such films may thus become established on any organic or inorganic surface substrate where planktonic microorganisms prevail in a water-based solution. In dental contexts, a well-known and extensively studied biofilm structure is established during the attachment of bacteria to teeth to form dental plaque. Here, bacteria floating in saliva (planktonic organisms) serve as the primary source for the organization of this specific biofilm (30). However, in endodontics the biofilm concept has far gained limited attention. It has been discussed mainly within the framework of bacterial appearances on root tips of teeth with non-vital pulps. Such bacterial aggregations have been thought to be the cause of therapy-resistant apical periodontitis. Although not described in great detail, bacterial condensations on the walls of infected root canals have been observed. Anti-microbial agents have often been developed and optimized for their activity against fast growing, dispersed populations containing a single microorganism. However, microbial communities grown in biofilms are remarkably difficult to eradicate with anti-microbial agents and microorganisms in mature biofilms can be notoriously resistant for reasons that have yet to be adequately explained (30). There are reports showing that microorganisms grown in biofilms could be two- to 1000-fold more resistant than the corresponding planktonic form (31). Spratt *et al.* (32) evaluated the effectiveness of NaOCl (2.25%), 0.2% CHX, 10% povidone iodine, 5 ppm colloidal silver and phosphate buffered solution ((PBS) as control) against monoculture biofilms of five root canal isolates including *P. intermedia*, *Peptostreptococcus micros*, *Streptococcus intermedius*, *F. nucleatum*, and *E. faecalis*. Results showed that NaOCl was the most effective anti-microbial followed by the iodine solution. Clegg *et al.* (33) evaluated the effectiveness of three concentrations of sodium hypochlorite (6%, 3%, and 1%), 2% CHX and BioPure MTAD on apical dentine biofilms* in vitro*. Results showed that 6% NaOCl and 3% NaOCl were capable of disrupting and removing the biofilm; 1% NaOCl and 1% NaOCl followed by MTAD were capable of disrupting the biofilm, but not eliminating bacteria; 2% CHX was not capable of disrupting the biofilm. Viable bacteria could not be cultured from specimens exposed to 6% NaOCl, 2 % CHX, or 1% NaOCl followed by BioPure MTAD.

Dunavant *et al.* (34) evaluated the efficacy of 6% NaOCl, 1% NaOCl, Smear Clear™, 2% CHX, REDTA, and BioPure™ MTAD™ against *E. faecalis* biofilms using a novel *in vitro* testing system. Biofilms grown in a flow cell system were submerged in test irrigants for either 1 or 5 minutes. There was a significant relationship between test agent and percentage kill of the biofilm bacteria. No significant relationship between time and kill percentage was found. The percentage kill of the biofilms bacteria was: 6% NaOCl (>99.99%), 1% NaOCl (99.78%), Smear Clear™ (78.06%), 2% CHX (60.49%), REDTA (26.99%), and BioPure™ MTAD™ (16.08%). There was a significant difference between 1% and 6% NaOCl, and all other agents. Therefore, both 1% NaOCl and 6% NaOCl were more efficient in eliminating *E. faecalis* biofilm than the other solutions tested. In another study, Lima *et al.* (35) assessed the effectiveness of CHX- or antibiotics (clindamycin with metronidazole)-based medications in eliminating *E. faecalis *biofilms. One-day and three-day biofilms of *E. faecalis* were used. Each biofilm-containing membrane was thoroughly covered with 1 ml of the test medications and incubated for 1 day at 37°C. Treated biofilms were then aseptically transferred to vials containing a neutralizing agent in saline solution and vortexed. Suspensions were 10-fold diluted, seeded onto *Mitis salivarius* agar plates, and the colony-forming units counted after 48 h of incubation. There were significant differences between the formulations tested. The association of clindamycin with metronidazole significantly reduced the number of cells in 1-day biofilms. However, of all medications tested, only 2% CHX-containing medications were able to thoroughly eliminate most of both 1-day and 3-day *E. faecalis* biofilms.

## Substantivity

CHX has a unique feature in that dentine medicated with it acquires antimicrobial substantivity. The positively-charged molecules of CHX can adsorb onto dentine and prevent microbial colonization on the dentine surface for some time beyond the actual medication period (8).

Antimicrobial substantivity of CHX has been assessed in several periodontal and endodontic studies. In an *in vivo* periodontal study, Stabholz *et al*. (36) evaluated the substantivity effect on human root surface after in situ subgingival irrigation with tetracycline HCL and CHX. They found that the substantivity of tetracycline 50 mg/ml was significantly greater than CHX for 12 days and greater than saline for 16 days.

In an *in vitro* study, White *et al*. (37) evaluated the antimicrobial substantivity of 2%CHX solution as an endodontic irrigation. Findings showed that substantivity lasted for 72h. In an *in vivo* study to evaluate the substantivity of 2% CHX solution, Leonardo *et al*. (38) evaluated the antimicrobial substantivity of 2% CHX used as a root canal irrigating solution in teeth with pulp necrosis and radiographically visible chronic periapical lesions. They found that CHX prevents microbial activity with residual effects in the root canal system for up to 48 h. However, some other studies revealed the substantivity of CHX for longer periods. Khademi *et al*. (39) found that 5-min treatment with 2% CHX solution induced substantivity for up to 4 weeks. Rosenthal *et al*. (40) evaluated the substantivity of CHX within the root canal system after 10-min treatment with 2% CHX solution. They found that CHX was retained in the root canal dentine in anti-microbially effective amounts for up to 12 weeks. Antimicrobial substantivity depends on the number of CHX molecules available to interact with the dentine. Therefore, medicating the canal with a more concentrated CHX preparation should result in increased resistance to microbial colonization. Recently, antibacterial substantivity of three concentrations of CHX solution (4%, 2% and 0.2%) after 5-min have been evaluated. Results revealed a direct relationship between the concentration of CHX and its substantivity (41). On the contrary, Lin *et al*. (42) attributed the substantivity of CHX to absorb the medication to dentine during the first hour and stated that it is only after the saturation point after the first hour that the antimicrobial capability of CHX increases with time. Furthermore, Komorowski *et al. *(43) revealed that 5-min CHX treatment did not induce substantivity, and dentine should be treated with CHX for 7 days.

## Buffering effect of dentine on CHX

Root canal milieu is a complex mixture of a variety of organic and inorganic compounds. Hydroxyapatite, the main component of dentine, is the major representative of inorganic components present. In addition, inflammatory exudate, entering the apical root canal in purulent infections, is rich in proteins such as albumin. The relative importance of the various organic and inorganic compounds in the inactivation of root canal disinfectants have been studied restrictively (44). Difficulties in designing experiments that will give reliable and comparable data were one of the great challenges for researchers for many years. Ultimately, Haapasalo *et al.* (44) introduced a new dentine powder model for studying the inhibitory effect of dentine on various root canal irrigants and medicaments. Haapasalo *et al.* (44) reported that 0.05% CHX acetate killed greater than 99.9% of *E. faecalis* cells within one hour when dentine was not present. Addition of dentine (18% w/v) totally prevented killing of the bacteria during the first hour. However, at 24 hours all bacteria were killed in both groups. Further, they incubated CHX together with dentine for 1 hour and 24 h. Pre-incubation with dentine slightly weakened the long term effect of CHX, and after 24 h of incubation with bacteria less than 0.5% of the cells (*E. faecalis*) were still viable. Despite strong inhibition of calcium hydroxide, it had only a limited effect on the antibacterial activity of CHX, because approximately 95% of the* E. faecalis* cells were killed within 1 h of incubation with 0.05% in the presence of 18% (w/v) hydroxyapatite. Lower amounts of hydroxyapatite failed to show any detectable inhibition of CHX activity (45). They also found that bovine serum albumin (BSA) strongly inhibited the antibacterial activity of CHX (0.05%). This indicates that periapical inflammatory exudate entering the root canal as a greater threat to the activity of CHX than the dentine walls. In another study, Portenier *et al.* (46) assessed the antibacterial activity of CHX on *E. faecalis* in the presence of dentine, dentine matrix, dentine pretreated by EDTA and citric acid, collagen, and heat-killed cells of *E. faecalis* and *Candida albicans. *Dentine matrix and heat-killed microbial cells were the most effective inhibitors of CHX, whereas dentine pretreated by citric acid or EDTA showed only slight inhibition. Inhibitory effect of dentine and BSA on the antibacterial activity of CHX was assessed in another study (47). The presence of dentine or BSA caused a marked delay in killing of *E. faecalis*. The inhibitory effect of BSA on the antibacterial activity of CHX has been confirmed recently by Sassone *et al.* (48). Taken together, it seems that dentine, dentine components (HA and collagen), killed microorganisms and inflammatory exudates in the root canal system reduce or inhibit the antibacterial activity of CHX.

## Tissue solubility of CHX

Several studies have been conducted in search for an irrigant that meets four major properties: antimicrobial activity, non-toxicity to periapical tissues, water solubility and capacity to dissolve organic matter. Therefore, an ideal irrigant should dissolve the organic matter inside the root canal system. Grossman and Meiman (49) demonstrated the importance of the solvent ability of an endodontic irrigant and emphasized that the elimination of pulp tissue from the root canal was important for the ultimate success of root canal treatment. Moorer and Wesselink (50) showed that tissue dissolution was dependent on three factors: frequency of agitation, amount of organic matter in relation to amount of irrigant in the system and surface area of tissue that was available. Okino *et al.* (51) evaluated the tissue dissolving ability of 0.5, 1.0 and 2.5% sodium hypochlorite; 2% aqueous solution of CHX digluconate; 2% chlorhexidine digluconate gel (Natrosol™); and distilled water as control. Bovine pulp fragments were weighed and placed in contact with 20 mL of each tested substance in a centrifuge at 150 rpm until total dissolution. Dissolution speed was calculated by dividing pulp weight by dissolution time. Distilled water and both solutions of CHX did not dissolve the pulp tissue within 6 h. Mean dissolution speeds for 0.5, 1.0 and 2.5% sodium hypochlorite solutions were 0.31, 0.43 and 0.55 mg min^-1^, respectively. The solvent ability of CHX solutions was similar to that of distilled water. In another study, Naenni *et al.* (52) assessed the necrotic tissue dissolution capacity of 1% NaOCl (wt/vol), 10% CHX, 3% and 30% hydrogen peroxide, 10% peracetic acid, 5% dichloroisocyanurate (NaDCC), and 10% citric acid. Standardized necrotic tissue samples obtained from pig palates were incubated in these solutions, and their weight loss was measured over time. None of the test solutions except sodium hypochlorite had any substantial tissue dissolution capacity. It was concluded that this might be important when considering the use of irrigants other than NaOCl.

## CHX and Calcium hydroxide

CHX is a cationic biguanide that its optimal antimicrobial activity is achieved within a pH range of 5.5 to 7.0 (8). Therefore, it seems that alkalinizing pH by adding calcium hydroxide to CHX precipitates CHX molecules and decreases its effectiveness. However, it has been demonstrated that the alkalinity of calcium hydroxide in the mixture remained unchanged. Therefore, the usefulness of mixing CH with CHX has still remained unclear and is under controversy (8).

When used as an intracanal medicament, CHX was more effective than calcium hydroxide (CH) in eliminating *E. faecalis *from inside dentinal tubules (8). In a study by Almyroudi *et al*. (53), all of the CHX formulations used, including a CHX/CH 50:50 mix, were efficient in eliminating *E. faecalis *from the dentinal tubules with a 1% CHX gel working slightly better than the other preparations. These findings were corroborated by Gomes *et al*. (54) in bovine dentine and Schafer and Bossmann (55) in human dentine where 2% CHX gel had greater activity against *E. faecalis*, followed by CHX/CH and then CH used alone.

In a study using agar diffusion, Haenni *et al*. (56) could not demonstrate any additive antibacterial effect by mixing CH powder with 0.5% CHX. In fact, they showed that the CHX had a reduced antibacterial action. However, CH did not lose its antibacterial properties in such a mixture. This may be due to the deprotonation of CHX at a pH greater than 10, which reduces its solubility and alters its interaction with bacterial surfaces as a result of the altered charge of the molecule. In an *in vitro *study using human teeth, Ercan *et al*. (57) showed 2% CHX gel was the most effective agent against *E. faecalis *inside dentinal tubules, followed by a CH/2% CHX mix, whilst CH alone was totally ineffective, even after 30 days. The 2% CHX gel was also significantly more effective than the CH/2% CHX mix against *C. albicans *at seven days, although there was no significant difference at 15 and 30 days. CH alone was completely ineffective against *C. albicans*. In another *in vivo *study using primary teeth, a 1% CHX gluconate gel, both with and without CH, was more effective against *E. faecalis *than CH alone within a 48-hour period (58).

Schafer and Bossmann (55) reported that 2% CHX gluconate was significantly more effective against *E. faecalis *than a CH used alone, or a mixture of the two. This was also confirmed by Lin *et al*. (59) although in a study by Evans *et al*. (60) using bovine dentine, 2% CHX with CH was shown to be more effective than CH in water. In an animal study, Lindskog *et al*. (61) reported that teeth dressed with CHX for 4 w had reduced inflammatory reactions in the periodontium (both apically and marginally) and less root resorption. Waltimo *et al*. (26) reported that 0.5% CHX acetate was more effective at killing *C. albicans *than saturated CH, while CH combined with CHX was more effective than CH used alone. The high pH of CH was unaffected when combined with CHX in this study.

## CHX and coronal leakage

Due to its antimicrobial substantivity, it seems that CHX preparations delay microleakage into the root canal. In an *in vitro* study, Gomes *et al.* investigated the time required for recontamination of coronally unsealed canals medicated with either calcium hydroxide, 2% CHX gel or with a combination of both (62). The canals without coronal seal, but medicated with CHX, showed recontamination after an average time of 3.7 d; the group with Ca(OH)_2_ after 1.8 d and the group with CHX + Ca(OH)_2_ after 2.6 d. The canals medicated with CHX + IRM showed recontamination within 13.5 days; the group with Ca(OH)_2_ + IRM after 17.2 d and the group with CHX+ Ca(OH)_2_ +  IRM after 11.9 d. The group with no medication, but sealed with IRM, showed recontamination after an average time of 8.7 d. There were statistically significant differences between the groups (*P* < 0.05). All groups without coronal seal were recontaminated significantly more quickly than those sealed with IRM, except those teeth coronally sealed but without medicament. The groups with intracanal medication and sealed were not significantly different from each other. Vivacqua-Gomes *et al****.*** (63) assessed *in vitro* coronal microleakage in extracted human teeth after root-canal treatment using 1% NaOCl, 1% NaOCl + 17% EDTA, 2% CHX gel, 2% CHX gel + 1% NaOCl, and distilled water. After root-canal filling, the teeth were incubated at 37 °C for 10 days followed by 10 days immersion in human saliva and an additional 10 days in India ink. The teeth were cleared and maximum dye penetration was determined digitally in millimeters. Results revealed that least leakage occurred with 1% NaOCl + 17% EDTA and 2% CHX gel. NaOCl, distilled water and 2% CHX gel + 1% NaOCl gave increased leakage with a significant difference compared to NaOCl + 17% EDTA and 2% CHX gel, and compared to one another. On the other hand, some studies showed that viscous irrigants, including those containing chlorhexidine gluconate, were less soluble substances, leaving residues on the root-canal surfaces which impaired final obturation. Lambrianidis *et al.* (64) investigated the efficiency of removing calcium hydroxide/CHX gel, Ca(OH)_2_/CHX solution and Ca(OH)_2_/saline pastes with the use of instrumentation and irrigation with NaOCl and EDTA solutions. None of the techniques used in this study removed the inter-appointment root canal medicaments effectively (64). Overall, Ca(OH)_2_/CHX (gel) paste was associated with significantly larger amount of residue, whereas Ca(OH)_2_/CHX (solution) paste was associated with less residue than the other two medicaments. Taken together due to its substantivity, CHX as an intracanal medicament/irrigant delays recontamination of the root canal system via coronal route.

## CHX and apical leakage

Marley *et al.* (65) assessed the effect of 0.12% CHX gluconate as an endodontic irrigants on the apical seal of obturated root canals using three different sealers (Roth's 811, AH26, and Sealapex). At 90 and 180 d after obturation, apical leakage was measured by the fluid filtration method. The results showed no significant difference in seal related to the irrigant at both the 90- and 180-day observation periods. Also, the same group reported that at long-term periods (270 and 360 d), CHX gluconate irrigant did not adversely affect the apical seal of the root canal cements (66). Wuerch *et al*. (67) investigated the effect of CHX gel and CH on the apical seal of the root-canal system. Results demonstrated that 2% CHX gel and calcium hydroxide paste did not adversely affect the apical seal of the root-canal system. These findings confirmed by Engel *et al.* (68). Overall, it seems that medication and/or irrigation with CHX does not adversely affect the apical seal of the root canal.

## CHX and mineral trioxide aggregate (MTA)

MTA is marketed in gray colored and white colored preparations: both are75% Portland cement, 20% bismuth oxide and 5% gypsum by weight. MTA is a hydrophilic powder which requires moisture for setting. Traditionally, MTA powder is mixed with supplied sterile water in a 3:1 powder/liquid ratio. Different liquids have been suggested to be mixed with MTA powder such as lidocaine anesthetic solution, sodium hypochlorite and CHX (69). Stowe *et al.* (70) determined the effect of the substitution of 0.12% CHX for sterile water as a mixing agent on the antimicrobial activity of white MTA. They found that substituting 0.12% CHX for water enhanced the antimicrobial activity of MTA. This finding was confirmed by Holt *et al.* (71). Hernandez *et al.* (72) compared the percentage of apoptotic cells and the cell cycle profile of fibroblasts and macrophages exposed to either MTA mixed with CHX, or exposed to MTA mixed with sterile water. Results showed that MTA specimens containing CHX induced apoptosis of macrophages and fibroblasts. In contrast, no change in the proportion of apoptotic cells was observed when sterile water was used to prepare the specimens. Cell cycle analysis showed that exposure to MTA/CHX decreased the percentage of fibroblasts and macrophages in S phase (DNA synthesis) as compared with exposure to MTA/water. On the other hand, Sumer *et al.* (73) examined the biocompa-tibility of MTA mixed with CHX histopatho-logically. They found that MTA/CHX surrounded by fibrous connective tissue, which indicated that it was well tolerated by the tissues. Yan *et al.* (74) found that CHX had no negative effect on the bond strengths of MTA-dentin in vitro. Kogan *et al.* (75) found that the MTA product prepared with CHX did not set. Furthermore, Holt *et al.* (71) found that MTA mixed with sterile water always had higher compressive strengths than MTA mixed with CHX. Shahi *et al.* (76) evaluated the sealing ability of white and gray MTA mixed with distilled water and 0.12% CHX when used as root-end filling materials. Results showed that CHX had no negative effect on the sealing ability of MTA. On the whole, it can be concluded that mixing MTA powder with CHX increases its antimicrobial activity but may have a negative effect on its mechanical properties.

## Toxicity of CHX

Results from a study on the cytotoxic effect of CHX on canine embryonic fibroblasts and *Staphylococcus aureus* showed that bactericidal concentrations of chlorhexidine were lethal to canine embryonic fibroblasts whilst non-cytotoxic concentrations allowed significant bacterial survival (77). In a study by Tatnall *et al.* (78), the cytotoxic effects of CHX, hydrogen peroxide and sodium hypochlorite were examined on cultured human fibroblasts, basal keratinocytes and a transformed keratino-cyte line (SVK 14 cells). At concentrations recommended for wound cleansing all agents produced 100% killing of all cell types. Comparison of the ED_50_ concentration for each agent on all cell types produced a ranking order of toxicity showing CHX to be the least toxic antiseptic agent.

Results from an *in vitro* study on the toxicity of CHX to human gingival cells showed that the toxic potency of chlorhexidine is dependent on the length of exposure and the composition of the exposure medium (79). Addition of fetal bovine serum, albumin, lecithin and heat-killed *Escherichia coli* reduced the cytotoxicity of CHX, presumably due to the binding of the cationic CHX to the negatively charged chemical moieties/sites of these components/ bacteria (79). These findings suggest that similar reactions within a root canal may reduce the potential of a cytotoxic reaction in the periapical tissues (80). Boyce *et al.* (80) found chlorhexidine (0.05%) uniformly toxic to both cultured human cells and microorganisms. Agarwal *et al.* (81) found that CHX rapidly disrupts the cell membrane of both crevicular and peripheral blood neutrophils at concentrations above 0.005% within 5 min, indicating that its inhibitory effect on neutrophil function is mostly due to its lytic properties. Yesilsoy *et al.* (82) assessed the short-term toxic effects of CHX in the subcutaneous tissue of guinea pigs and found a moderate inflammation present after 2 days, followed by a foreign-body granuloma formation at 2 w. Ribeiro *et al.* (83) evaluated the genotoxicity (potential damage to DNA) of formocresol, paramonochlorophenol, calcium hydroxide, and CHX against Chinese hamster ovary (CHO) cells. Results showed that none of the mentioned agents had any contribution to the DNA damage.

## Allergic reactions to CHX

Although sensitivity to CHX is rare, contact dermatitis is a common adverse reaction to CHX (84). Apart from that, CHX is liable to a number of rare side effects, such as desquamative gingivitis, discolouration of teeth and tongue or dysgeusia (distorted taste). Contact with conjunctiva can cause permanent damage, and accidental contact with the tympanum can cause ototoxicity (85). Various allergic reactions due to CHX have been described. Contact sensitivity to CHX was first reported by Calnan in 1962 (86). Today, CHX is known to elicit allergic contact dermatitis, including connubial contact dermatitis, generally after prolonged and repeated application (84). It can also cause contact urticaria, photosensitivity, fixed drug eruption and occupational asthma. People at particular risk of contact allergy are, apart from medical staff, patients with leg ulcers and leg eczema (84). Altogether, contact sensitivity to CHX seems to be rare. Some larger studies showed a sensitization rate of about 2% (87-89). Even rarer are reports of immediate anaphylactic reactions due to CHX. Ohtoshi (90) demonstrated IgE antibodies in the serum of patients with anaphylaxis due to CHX. Application of CHX to intact skin can cause immediate allergic reactions such as urticaria, Quincke's edema or dyspnea and very rarely severe anaphylactic reactions (91-92). Taken together, it is important to keep in mind this potential risk of CHX.

## Conclusion

**1- **CHX has a wide range of activity against both Gram positive / negative bacteria.

**2-** CHX is an effective antifungal agent especially against *C. albicans*.

**3- **The effect of CHX on microbial biofilms is significantly lesser than sodium hypochlorite.

**4- **CHX has antibacterial substantivity for up to 12 weeks.

**5- **It seems that dentine, dentine components 

(HA and collagen), killed microorganisms and inflammatory exudates in the root canal reduce or inhibit the antibacterial activity of CHX.

**6- **Tissue solubility of CHX is little to none.

**7- **Mixing CHX with calcium hydroxide may enhance its antimicrobial activity.

**8- **CHX may delay coronal leakage in endodontically treated teeth.

**9- **It seems that medication and/or irrigation with CHX does not adversely affect the apical seal of the root canal.

**10- **Mixing MTA with CHX increases the antimicrobial properties of MTA, but has adverse effects on its mechanical properties.

**11- **Biocompatibility of CHX is acceptable. In rare cases CHX may cause allergic reactions.
